# Adjuvant Chemotherapy for Patients With Chronic Kidney Disease: A Study on Treatment Adoption and Associated Factors

**DOI:** 10.1002/cam4.71237

**Published:** 2025-09-09

**Authors:** Taisuke Ishii, Tomone Watanabe, Yuichi Ichinose, Hiroyuki Mano, Takahiro Higashi

**Affiliations:** ^1^ Division of Health Services Research, Institute for Cancer Control National Cancer Center Tokyo Japan; ^2^ Institute for Global Health Policy Research National Center for Global Health and Medicine Tokyo Japan; ^3^ Department of Public Health and Policy, Graduate School of Medicine The University of Tokyo Tokyo Japan

**Keywords:** adjuvant chemotherapy, chemotherapy regimen, chronic kidney disease, comorbidity, neoplasms

## Abstract

**Introduction:**

Patients with chronic kidney disease (CKD) face unique challenges in cancer treatment, including the need for chemotherapy dose adjustments and avoiding nephrotoxic agents, often leading to less aggressive treatment. However, little is known about the real‐world administration of adjuvant chemotherapy for patients with CKD. In this study, we aimed to investigate the prevalence of adjuvant chemotherapy in patients with CKD and to explore factors influencing chemotherapy use.

**Methods:**

We retrospectively analyzed data from the Diagnosis Procedure Combination survey and hospital‐based cancer registry in Japan. Adult patients diagnosed with colon, gastric, breast, or non‐small‐cell lung cancer who underwent curative surgery from January 2016 to December 2019 were included. CKD was identified based on International Classification of Diseases, 10th revision codes, and CKD‐related medication prescriptions. The primary outcome was the proportion of patients receiving adjuvant chemotherapy, and secondary outcomes were regimen details.

**Results:**

A total of 109,875 patients were included in the study, 4.5% (4953) of whom had CKD. Patients with CKD were older and had a higher prevalence of comorbidities. A smaller proportion of patients with CKD received adjuvant chemotherapy (41.7% vs. 64.5%, *p* < 0.001). CKD was independently associated with lower odds of receiving adjuvant chemotherapy (odds ratio: 0.51, 95% confidence interval: 0.48–0.55). Patients with CKD were also less likely to receive standard chemotherapy regimens or those requiring dose adjustments.

**Conclusions:**

Patients with CKD received adjuvant chemotherapy less frequently, likely owing to concerns about kidney toxicity and dose adjustments. Individualized treatment approaches are needed to optimize outcomes for this population.

## Graphical abstract

Among all patients, those with CKD received adjuvant chemotherapy less frequently than those without CKD.

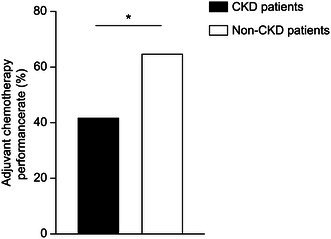

## 1 Introduction

Chronic kidney disease (CKD) is a prevalent comorbidity among patients with cancer and is significantly associated with poorer outcomes [1, 2, 3, 4]. CKD is recognized as an independent risk factor for cancer mortality and contributes to the poor overall prognosis in this population. The management of cancer in patients with CKD presents unique challenges owing to the impact of impaired kidney function on treatment decisions, particularly when administering chemotherapy. Impaired kidney function often necessitates dose adjustments or the selection of alternative chemotherapeutic agents to avoid toxicity, complicating treatment optimization [5, 6, 7].

Along with these challenges, concerns are growing that patients with cancer and CKD may not be receiving adequate oncological care [8, 9]. Some experts suggest that the presence of CKD necessitates more conservative treatment approaches, resulting in insufficient cancer therapy. However, this hypothesis remains largely speculative because of the lack of substantial evidence. The current state of chemotherapy use, including the prevalence of adjuvant chemotherapy, in patients with cancer and CKD is poorly understood, as few studies have been performed on this population.

Understanding the real‐world patterns of chemotherapy administration in patients with cancer and CKD is crucial for the improvement of treatment protocols and outcomes. To this end, this study was conducted with the aim to investigate the percentage of patients with CKD who are administered adjuvant chemotherapy and to explore the factors that may influence these treatment decisions. By shedding light on current practices, we sought to contribute valuable evidence to guide more effective cancer treatment strategies for this vulnerable patient population.

## 2 Materials & Methods

### 2.1 Study Design and Population

In this retrospective study, we included adult patients who underwent surgery for colon cancer, gastric cancer, non‐small‐cell lung cancer (NSCLC), or breast cancer and were newly diagnosed from January 2016 to December 2019. Patient data were obtained from the Diagnosis Procedure Combination (DPC) survey, which provides detailed insurance claims data, and from hospital‐based cancer registries (HBCRs). Only patients who had already undergone surgery were included, and those with preexisting metastatic disease at diagnosis were excluded. To ensure the inclusion of patients eligible for adjuvant chemotherapy, the study was limited to those with specific pathological stages for each type of cancer, as follows: colon cancer, pathological stage (pStage) III; gastric cancer, pStage II and III (except pT1 and pT3N0); NSCLC, pStage II and IIIA; and breast cancer, pStage III (except pT3N1). The study cohort was divided into two groups: patients with and those without CKD.

### 2.2 Outcome Measures

The primary outcome measure of this study was the proportion of patients with CKD receiving adjuvant chemotherapy compared with that of patients without CKD. Additionally, we aimed to identify factors associated with the administration of adjuvant chemotherapy in patients with CKD by using a multivariable logistic regression model. The secondary outcomes were as follows: the proportion of patients receiving standard chemotherapy regimens versus that of patients receiving non‐standard regimens, detailed analysis of non‐standard regimens used by patients with CKD, and the rate of patients requiring adjuvant chemotherapy dose adjustment based on kidney function. In describing the details of non‐standard regimens, we focused on colon and gastric cancer because of the large samples.

### 2.3 Definitions

CKD was defined as the presence of International Classification of Diseases, 10th revision (ICD‐10) codes associated with CKD in the linked dataset and/or the prescription of medications commonly used to manage CKD. These medications were phosphate binders, erythropoiesis‐stimulating agents, potassium binders, and carbonaceous adsorbents [10].

Based on prior studies, adjuvant chemotherapy was defined as postoperative chemotherapy administered within 3 months of surgery for colon or gastric cancer and within 6 months for NSCLC and breast cancer [11]. According to clinical guidelines [12, 13, 14, 15], standard chemotherapy regimens were categorized as follows: for colon cancer, the regimens were 5‐fluorouracil (5‐FU) plus oxaliplatin, 5‐FU alone, capecitabine plus oxaliplatin, tegafur‐uracil (UFT), and tegafur‐gimeracil‐oteracil (S‐1). For gastric cancer, the standard regimens were S‐1 plus oxaliplatin, capecitabine plus oxaliplatin, and S‐1 plus docetaxel. The standard treatment regimens for NSCLC were cisplatin (CDDP) plus pemetrexed (PEM), CDDP plus gemcitabine (GEM), CDDP plus docetaxel, CDDP plus vinorelbine, CDDP plus etoposide, carboplatin (CBDCA) plus PEM, CBDCA plus GEM, CBDCA plus paclitaxel, and UFT. For breast cancer, the standard treatments were combinations of doxorubicin or epirubicin with cyclophosphamide, taxanes, or doxorubicin; epirubicin combined with cyclophosphamide and 5‐FU; docetaxel combined with cyclophosphamide; and cyclophosphamide combined with methotrexate and 5‐FU.

Additionally, based on previous studies and Japanese guidelines [16, 17, 18, 19], we identified chemotherapy regimens that required dose adjustments for patients with CKD according to the first regimen administered after surgery. Doses are typically adjusted to minimize toxicity while maintaining therapeutic efficacy, with adjustments based on creatinine clearance or the estimated glomerular filtration rate (eGFR). In this study, comorbidities such as cardiovascular disease, diabetes mellitus, and liver disease were considered factors that might have influenced the administration of adjuvant chemotherapy. These conditions were identified using ICD‐10 codes related to the Charlson Comorbidity Index [20, 21].

### 2.4 Data Sources and Study Approval

The data for this study were obtained from a dataset linked to both the DPC survey and HBCRs. HBCRs form a part of the mandatory cancer‐reporting system for all cancer care provided at key hospitals designated by the Ministry of Health, Labor and Welfare in Japan [22]. Additionally, non‐designated hospitals with similar roles in their communities can voluntarily participate in such an HBCR. The HBCRs capture information on approximately 70%–80% of all patients with cancer in Japan [23]. They are governed by national regulations and are used to record clinical data, such as age at diagnosis, sex, cancer type, clinical and pathological cancer stages, tumor‐node‐metastasis classification, and histopathological results (coded according to the International Classification of Diseases for Oncology, 3rd edition); and the treating hospital's name. We collected DPC survey data for patients registered in the HBCR system from October of the year prior to diagnosis to March of the second year following diagnosis. In this study, DPC survey data were available for approximately 80% of the patients registered in the HBCR system.

This study was approved by the Institutional Review Board of the National Cancer Center, Japan (approval number 2022‐133) and was conducted in accordance with the Declaration of Helsinki. In accordance with Japanese personal information protection laws, the institutional review board approved a waiver of written informed consent and the use of an opt‐out approach for this observational study, whereby data from eligible patients were included unless they declined participation.

### 2.5 Statistical Analysis

Descriptive statistics were used to summarize the patient characteristics, including age, sex, cancer type, and CKD status. Continuous variables were analyzed using either Student's *t*‐test or the Mann–Whitney *U* test, depending on the data distribution. Categorical variables were compared using the chi‐squared test. A logistic regression model was used to identify the factors associated with adjuvant chemotherapy administration in patients with CKD. The variables selected for the regression model were based on clinical relevance: age, sex, cancer type, cancer stage, comorbidities, activities of daily living (ADLs) based on the Barthel Index [24], and CKD status.

A sensitivity analysis was performed to compare postoperative adjuvant chemotherapy rates among facilities capable of managing patients with severe CKD, excluding those that did not treat such patients. This analysis included only patients treated at facilities in which at least one of the patients underwent hemodialysis at least once during the study period according to the DPC data.

All statistical analyses were performed using Stata version 15.1 (Stata Corp LP, College Station, TX, USA), and a two‐sided *p* value of less than 0.05 was considered statistically significant.

## 3 Results

### 3.1 Patient Characteristics

Our study cohort comprised 109,875 patients who underwent curative surgery for colon cancer, gastric cancer, NSCLC, or breast cancer at 685 hospitals in Japan. Overall, 4953 (4.5%) patients were also diagnosed with CKD. As shown in Table 1, patients with CKD were generally older and exhibited lower degrees of functional independence than those without CKD. Additionally, the CKD group had a higher prevalence of comorbidities, reflecting the more complex health profiles of this group.

**TABLE 1:** Patient characteristics stratified according to CKD status.

Characteristics	Total (*N* = 109,875)	Patients with CKD (*n* = 4953)	Patients without CKD (*n* = 104,922)	*P* value
Age in years, mean (SD, min–max) (years)	70 (12, 20–105)	75 (9, 25–98)	70 (12, 20–105)	< 0.001
< 65, *n* (%)	29,223 (26.6)	641 (12.9)	28,582 (27.2)	< 0.001
65–74, *n* (%)	38,963 (35.5)	1687 (34.1)	37,276 (35.5)	
≥ 75, *n* (%)	41,689 (37.9)	2625 (53.0)	39,064 (37.2)	
Sex, *n* (%) (female)	48,641 (44.3)	1469 (29.7)	47,172 (45.0)	< 0.001
Cancer type, *n* (%)				< 0.001
Colon cancer	58,627 (53.4)	2607 (52.6)	56,020 (53.4)	
Gastric cancer	28,991 (26.4)	1422 (28.7)	27,569 (26.3)	
NSCLC	12,972 (11.8)	722 (14.6)	12,250 (11.7)	
Breast cancer	9285 (8.5)	202 (4.1)	9083 (8.7)	
Adjuvant chemotherapy, *n* (%)	69,725 (63.5)	2067 (41.7)	67,658 (64.5)	< 0.001
Postoperative length of stay, median (IQR) (days)	11 (8–16)	14 (10–21)	11 (8–16)	< 0.001
Duration of adjuvant chemotherapy, median (IQR) (days)	154 (98–229)	147 (65–229)	154 (99–228)	< 0.001
Barthel Index score, *n* (%)				< 0.001
100	97,607 (88.8)	4045 (81.7)	93,562 (89.2)	
60–95	8035 (7.3)	596 (12.0)	7439 (7.1)	
< 60	4233 (3.9)	312 (6.3)	3921 (3.7)	
Comorbidity, *n* (%)	34,333 (31.2)	2947 (59.5)	31,386 (29.9)	< 0.001
Cardiovascular disease	15,297 (13.9)	1411 (28.5)	13,886 (13.2)	< 0.001
Diabetes mellitus	20,948 (19.1)	2058 (41.6)	18,890 (18.0)	< 0.001
Liver disease	4007 (3.6)	238 (4.8)	3769 (3.6)	< 0.001
Hospital type, *n* (%)				< 0.001
Non‐designated hospital	25,118 (22.9)	1287 (26.0)	23,831 (22.7)	
Designated hospital	84,757 (77.1)	3666 (74.0)	81,091 (77.3)	
Year of diagnosis, *n* (%)				< 0.001
2016	24,930 (22.7)	990 (20.0)	23,940 (22.8)	
2017	24,108 (21.9)	1038 (21.0)	23,070 (22.0)	
2018	29,971 (27.3)	1436 (29.0)	28,535 (27.2)	
2019	30,866 (28.1)	1489 (30.1)	29,377 (28.0)	

Abbreviations: CKD, chronic kidney disease; IQR, interquartile range; max, maximum; min, minimum; NSCLC, non‐small‐cell lung cancer; SD, standard deviation.

### 3.2 Proportion of Adjuvant Chemotherapy and Factors Related to Administration

**FIGURE 1:**
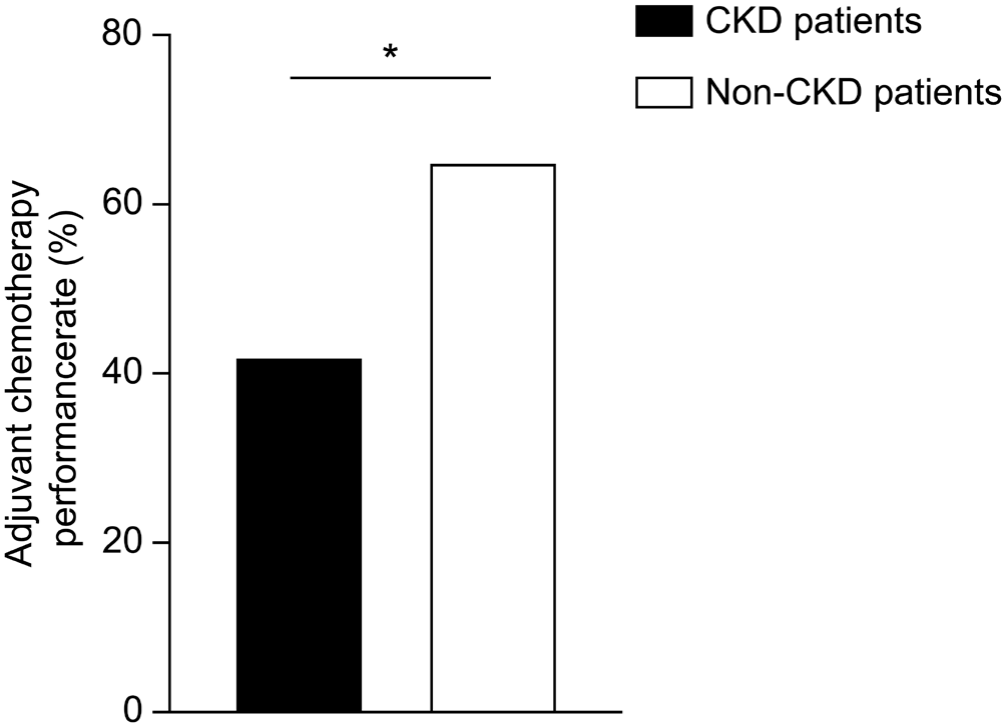
Differences in the administration of adjuvant chemotherapy between patients with cancer and CKD and those without CKD. Among all patients (*n* = 109,875), those with CKD received adjuvant chemotherapy less frequently than those without (41.7% vs. 64.5%) *p < 0.001. CKD, chronic kidney disease.

Overall, the proportion of patients receiving adjuvant chemotherapy was 41.7% among those with CKD and 64.5% among those without CKD (*p* < 0.001) (Figure 1). Table 2 highlights the key factors associated with adjuvant chemotherapy administration. CKD was associated with a significantly reduced likelihood of receiving chemotherapy (odds ratio [OR]: 0.51, *p* < 0.001) after adjusting for possible confounders. Age was also a strong factor, with patients aged 65–75 (OR: 0.62) and those aged older than 75 (OR: 0.15) being less likely to receive treatment than those aged < 65 (both *p* < 0.001). Lower Barthel Index scores, indicating reduced functional independence, were associated with a lower likelihood of chemotherapy (OR: 0.32 for 60–95, 0.09 for < 60, reference: 100, both *p* < 0.001). Comorbidities (OR: 0.81) and treatment at designated hospitals (OR: 0.92) were also significantly related to a lower likelihood of chemotherapy administration.

**TABLE 2:** Factors related to the performance of adjuvant chemotherapy in all patients (*N* = 109,875).

Characteristics	Unadjusted odds ratio (95% CI)	*P* value	Adjusted odds ratio (95% CI)^a^	*P* value
CKD				
No	Reference		Reference	
Yes	0.39 (0.37–0.42)	< 0.001	0.51 (0.48–0.55)	< 0.001
Age (years)				
< 65	Reference		Reference	
65–74	0.56 (0.54–0.58)	< 0.001	0.62 (0.60–0.65)	< 0.001
≥ 75	0.12 (0.11–0.12)	< 0.001	0.15 (0.14–0.15)	< 0.001
Sex				
Male	Reference		Reference	
Female	1.03 (1.00–1.05)	0.04	1.04 (1.01–1.07)	0.005
Barthel Index score				
100	Reference		Reference	
60–95	0.21 (0.20–0.22)	< 0.001	0.32 (0.31–0.34)	< 0.001
< 60	0.06 (0.06–0.07)	< 0.001	0.09 (0.09–0.10)	< 0.001
Comorbidity				
No	Reference		Reference	
Yes	0.59 (0.58–0.61)	< 0.001	0.81 (0.78–0.83)	< 0.001
Hospital type				
Non‐designated	Reference		Reference	
Designated	1.06 (1.03–1.09)	< 0.001	0.92 (0.89–0.95)	< 0.001

Abbreviations: CI, confidence interval; CKD, chronic kidney disease.

^a^ Adjusted for age at diagnosis, sex, Barthel Index, comorbidities, and hospital type.

### 3.3 Administration of Standard Regimens

**FIGURE 2:**
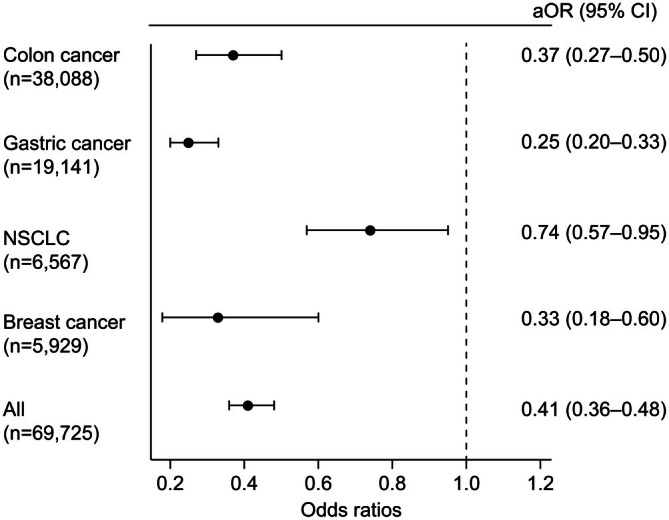
aOR for receiving standard regimen as adjuvant chemotherapy for each cancer type among patients with CKD compared with those without CKD. For colon cancer, gastric cancer, NSCLC, and breast cancer, we set the age at diagnosis, sex, comorbidity, the Barthel Index score, and hospital type as covariates in the logistic regression analysis for receiving a standard regimen as adjuvant chemotherapy. Among the respective cancer types, the odds of patients with CKD receiving a standard regimen was approximately 0.3–0.7 times that of patients without CKD (colon cancer: OR = 0.37, 95% CI = 0.27–0.50, *p* < 0.001; gastric cancer: OR = 0.25, 95% CI = 0.20–0.33, *p* < 0.001; NSCLC: OR = 0.74; 95% CI = 0.57–0.95, *p* = 0.02; and breast cancer: OR = 0.33; 95% CI = 0.18–0.60, *p* < 0.001). aOR, adjusted odds ratio; CKD, chronic kidney disease; CI, confidence interval; NSCLC, non‐small‐cell lung cancer; OR, odds ratio.

Figure 2 is a forest plot representing the association between receiving a standard chemotherapy regimen and having CKD across different cancer types. When all cancers were combined, the adjusted OR was 0.41 (0.36–0.48), revealing a general trend across all cancer types that patients with CKD were approximately 59% less likely to receive standard chemotherapy than those without. Although the OR varied across cancer types, the rate of chemotherapy administration was lower in patients with CKD than in patients without CKD for each cancer type.

### 3.4 Details of Non‐Standard Regimens

**FIGURE 3:**
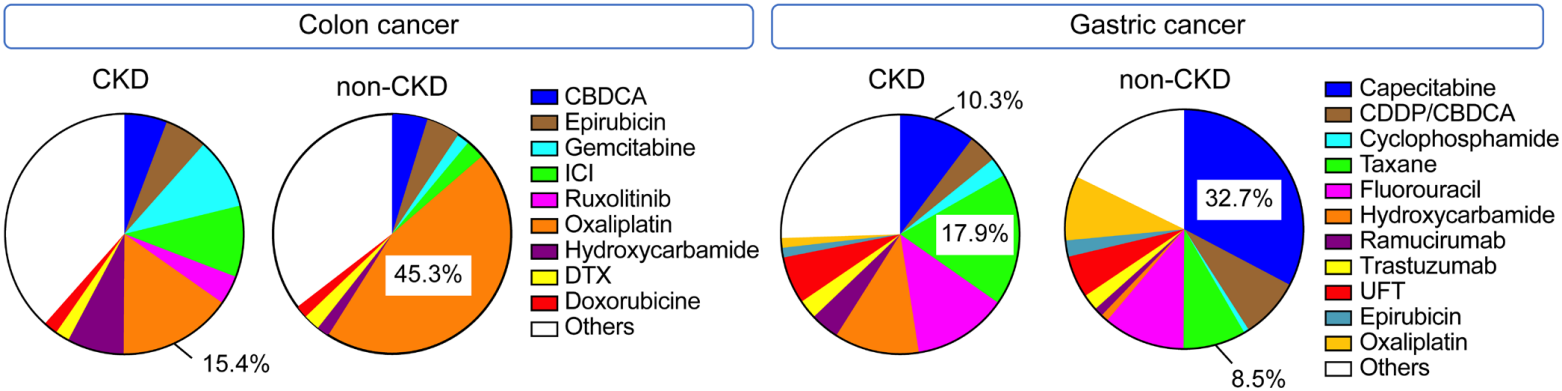
Details of non‐standard regimen administered to patients with colon or gastric cancer with or without CKD. Among patients with colon cancer receiving non‐standard regimens (*n* = 703), gemcitabine monotherapy and ICIs were frequently used, although oxaliplatin monotherapy was infrequently used by patients with CKD compared with that used by patients without CKD (gemcitabine: 9.6% vs. 1.7%; ICIs: 9.6% vs. 2.7%; oxaliplatin: 15.4% vs. 45.3%). Among patients with gastric cancer receiving non‐standard regimens (*n* = 675), taxane monotherapy was frequently used, although capecitabine monotherapy was infrequently used by patients with CKD compared with that used by patients without CKD (taxane: 17.9% vs. 8.5%; capecitabine: 10.3% vs. 32.7%). CBDCA, carboplatin; CDDP, cisplatin; CKD, chronic kidney disease; DTX, docetaxel; ICI, immune‐checkpoint inhibitor; UFT, tegafur‐uracil.

Figure 3 illustrates the differences in the use of non‐standard chemotherapy regimens between patients with colon or gastric cancer with or without CKD. For those with colon cancer, oxaliplatin monotherapy was used less frequently by patients with CKD (15.4%) than that used by patients without CKD (45.3%). For those with gastric cancer, capecitabine monotherapy was less commonly used by patients with CKD (10.3%) than that used by patients without CKD (32.7%). However, taxane monotherapy was more frequently administered to patients with gastric cancer and CKD than that administered to those without CKD. These results suggest that chemotherapy regimens were modified for patients with CKD, likely owing to concerns about kidney function, resulting in a shift away from standard treatments toward alternative options such as taxanes.

### 3.5 Administration of Regimens Requiring Dose Adjustment

Table 3 shows that compared with patients without CKD, those with CKD were less frequently administered chemotherapy agents requiring dose adjustments based on kidney function. This trend was consistent across all four cancer types.

**TABLE 3:** Proportion of patients who required dose adjustment based on kidney function among those who received adjuvant chemotherapy.

Characteristics	Patients with CKD		Patients without CKD		*P* value
	*n* (%)	Total	*n* (%)	Total	
All patients	1932 (93.5)	2067	65,854 (97.3)	67,658	< 0.001
Colon cancer	1023 (96.2)	1063	36,654 (99.0)	37,025	< 0.001
Gastric cancer	578 (93.1)	621	18,294 (98.8)	18,520	< 0.001
NSCLC	264 (88.9)	297	5843 (93.2)	6270	0.003
Breast cancer	67 (77.9)	86	5063 (86.7)	5843	0.02

Abbreviations: CKD, chronic kidney disease; NSCLC, non‐small cell lung cancer.

### 3.6 Sensitivity Analysis

Similar to the results of the primary analysis, the proportion of patients with CKD was 4.5%, and patients with CKD were older and had a higher prevalence of comorbidities than patients without CKD (Table S1). Additionally, in the multivariable analysis, patients with CKD were less likely to receive postoperative adjuvant chemotherapy than patients without CKD (OR: 0.51, 95% CI: 0.47–0.54, *p* < 0.001; Table S2).

## 4 Discussion

This study included 109,875 patients with cancer, 4.5% of whom had CKD. Adjuvant chemotherapy was less common in patients with CKD (41.7%) than that in patients without CKD (64.5%). CKD was associated with a significantly lower likelihood of receiving standard chemotherapy (OR: 0.41), as was a higher age, having comorbidities, and having a lower Barthel Index score. For non‐standard regimens, patients with colon cancer and CKD received oxaliplatin monotherapy less often than those without CKD, and patients with gastric cancer and CKD received capecitabine monotherapy less often than those without CKD, whereas patients with gastric cancer and CKD were more likely to receive taxane than those without CKD. In addition, chemotherapies requiring dose adjustments based on kidney function tend not to be selected as adjuvant chemotherapy for patients with cancer and CKD.

We demonstrated that the proportion of patients who received adjuvant chemotherapy was significantly lower among those with CKD than that among patients without, even after adjusting for age and other factors. This may be explained by the lack of robust evidence supporting the use of chemotherapy for patients with CKD. Many clinical trials exclude patients with impaired kidney function, often setting an eGFR threshold of 60 mL/min/1.73 m^2^ because CKD may increase the risk of adverse events, leading to treatment failure in clinical trials. This exclusion limits the available evidence on the efficacy and safety of chemotherapy among patients with CKD [25]. Patients with CKD reportedly have worse survival outcomes when they receive suboptimal cancer treatment, often driven by concerns about drug toxicity and altered pharmacokinetics owing to kidney impairment [8, 26]. Furthermore, CKD is recognized as a risk factor for kidney toxicity associated with several agents, including CDDP, PEM, and immune‐checkpoint inhibitors [27]. Additionally, for patients with CKD, an inaccurate assessment of kidney function may lead to inappropriate dosage determination, resulting in an increased likelihood of drug‐related adverse events [28]. Consequently, patients with CKD likely receive suboptimal treatment owing to the absence of clear clinical guidelines and concerns regarding adverse events. Although such concerns have been recognized, there has been a significant lack of studies that specifically detail the cancer treatments administered to patients with CKD. Using a real‐world dataset, our study revealed that patients with CKD were less likely to receive adjuvant chemotherapy than those without, despite their postoperative condition requiring adjuvant chemotherapy for curative treatment. Our results provide important evidence that visualizes and validates the hypothesis that patients with CKD have lower chemotherapy rates than those without, addressing a knowledge gap in the field.

Patients with CKD are generally older and have a higher prevalence of comorbidities than those without CKD [29, 30, 31]. Advanced age, the presence of comorbidities, and a decline in ADLs are significant factors that influence decision‐making regarding the administration of chemotherapy. Therefore, patients with CKD may be less likely to receive chemotherapy not only because of CKD itself but also because of related factors [25]. We investigated this point by conducting an analysis that accounted for age, sex, ADL score, and the presence of comorbidities such as diabetes and cardiovascular disease, assessing the relationship between CKD and adjuvant chemotherapy. Our study confirmed that age, ADL score, and the presence of comorbidities are associated with the decision not to administer adjuvant chemotherapy. However, CKD itself was also independently associated with a reduced likelihood of receiving adjuvant chemotherapy, even after adjusting for these factors. This result suggests that impaired kidney function may increase the complexity of cancer treatment, necessitating careful selection of chemotherapeutic agents and dose adjustments to minimize adverse effects. These factors, along with the need for scientific validation, may influence physicians' decision‐making processes.

The reasons for the lower use of standard regimens in patients with CKD are likely multifaceted. One contributing factor is the lack of clinical trial data for patients with CKD, as many trials exclude such patients based on kidney function criteria. In the absence of strong evidence, clinicians rely on expert recommendations, such as those from nephrology societies, which guide individualized treatments based on kidney function [19]. Studies have suggested that clinicians face substantial challenges in balancing the risks of kidney toxicity with the potential benefits of chemotherapy. For example, Hussain et al. conducted a phase I/II trial with GEM and a split dose of CDDP to investigate safe regimens for patients with bladder cancer and kidney impairment, as over 40% of patients with bladder cancer are considered ineligible for CDDP treatment owing to kidney impairment [32, 33]. Moreover, CBDCA has been investigated as a substitute for CDDP for patients with urogenital cancer [34]. This tendency is reflected in our study of non‐standard regimens, as patients with colon cancer and CKD were less likely to receive oxaliplatin monotherapy than those without CKD, and patients with gastric cancer and CKD were less likely to receive capecitabine and more likely to receive alternative agents, such as taxanes, which are primarily metabolized in the liver. Additionally, patients with CKD tended not to receive chemotherapy that requires dose adjustment based on kidney function compared with patients without CKD. These results suggest that clinicians modify treatment regimens for patients with CKD to minimize kidney toxicity, often opting for drugs that require fewer dose adjustments based on kidney function.

Given these complexities, individualized medical care for patients with CKD is essential [35]. Patients with CKD present unique challenges owing to altered drug metabolism and increased risks of treatment‐related toxicity. Therefore, cancer therapies should be tailored to patients' kidney function, cancer type, and overall health status to optimize outcomes. Personalized treatment plans that incorporate close monitoring of kidney function and corresponding adjustments to drug types and dosages should be prioritized to ensure that patients with CKD receive effective yet safe cancer care.

This study has several limitations. Owing to the nature of the dataset, CKD was identified based on ICD‐10 codes or the use of specific medications associated with CKD, which might have led to the disproportionate inclusion of CKD cases with higher severity. Therefore, patients with mild CKD might not have been classified as having CKD in this study, potentially underestimating the chemotherapy administration rate among patients without CKD. This limitation highlights the need for a more detailed classification of CKD severity in future research to provide a clearer picture of the treatment patterns in this population. Additionally, we could not evaluate the effectiveness of each regimen because of the limitations of the dataset. Whereas our study was focused on chemotherapy administration rates and patterns among patients with and those without CKD, the absence of data on treatment outcomes restricted our ability to assess the effectiveness of specific chemotherapy regimens in this population. To the best of our knowledge, this was the first study to demonstrate that patients with CKD are less likely to receive adjuvant chemotherapy or standard treatment regimens than those without. This indicates that regimens commonly used in the general population are not always suitable for patients with CKD. Thus, further studies with more granular clinical and survival data are needed to elucidate the various positive and negative effects of chemotherapeutic agents in patients with CKD. Prospective trials or larger datasets incorporating both kidney function and treatment outcomes would provide more comprehensive insights into the effectiveness of various chemotherapeutic regimens in patients with impaired kidney function. In the interim, rigorously analyzed real‐world evidence—incorporating robust confounding control, kidney‐specific safety outcomes, and hard endpoints such as overall survival—can inform guideline development and shared decision‐making. In clinical practice, dose adjustment and proactive renal monitoring can help balance oncologic benefit against nephrotoxicity and support the safe delivery of adjuvant chemotherapy in patients with CKD.

## 5 Conclusion

In conclusion, the results of this study suggest that clinicians modify treatment regimens to minimize kidney toxicity, but the lack of robust clinical trial data for patients with CKD leaves gaps in evidence‐based guidelines. The necessity for individualized treatment approaches for patients with CKD is clear, as they face unique risks and challenges in cancer therapy. Further research, including prospective trials of both treatment efficacy and kidney function outcomes, is crucial to optimize cancer care for this vulnerable population.

## Author Contributions

**Taisuke Ishii:** conceptualization (lead), data curation (lead), formal analysis (lead), funding acquisition (lead), methodology (lead), project administration (lead), visualization (lead), writing – original draft (equal). **Tomone Watanabe:** data curation (supporting), visualization (supporting), writing – original draft (equal). **Yuichi Ichinose:** data curation (supporting), investigation (supporting), writing – review and editing (equal). **Hiroyuki Mano:** supervision (equal), writing – review and editing (equal). **Takahiro Higashi:** conceptualization (supporting), supervision (equal), writing – review and editing (equal).

## Ethics Statement

This study was approved by the Institutional Review Board of the National Cancer Center, Japan (approval number 2022‐133) and was conducted in accordance with the Declaration of Helsinki.

## Consent

This study was an observational study and employed an opt‐out approach, using data only from patients who did not refuse data usage, in accordance with Japanese personal information protection laws and ethical guidelines.

## Conflicts of Interest

The authors declare no conflicts of interest.

## Supporting information


**Table S1:** Patient characteristics stratified according to CKD status in hospitals in which hemodialysis could be performed (sensitivity analysis).
**Table S2:** Factors related to the performance of adjuvant chemotherapy in all patients in hospitals in which hemodialysis could be performed (*n* = 103,076).

## Data Availability

The data that support the findings of this study are available on request from the corresponding author. The data are not publicly available due to privacy or ethical restrictions.
