# Supplementary material for: Adjuvant Chemotherapy for Patients With Chronic Kidney Disease: A Study on Treatment Adoption and Associated Factors
Source: Cancer Med. 2025 Sep 9;14(17):e71237. doi: 10.1002/cam4.71237 (PMC12418075; doi:10.1002/cam4.71237)
Supplement: Supplementary file 1 — Table S1: Patient characteristics stratified according to CKD status in hospitals in which hemodialysis could be performed (sensitivity analysis). Table S2: Factors related to the performance of adjuvant chemotherapy in all patients in hospitals in which hemodialysis could be performed (n = 103,076). [file CAM4-14-e71237-s001.docx]

**Supplementary Material**

**Supplementary Table 1**. Patient characteristics stratified according to CKD status in hospitals in which hemodialysis could be performed (sensitivity analysis)

| **Characteristics** | **Total**  **(n = 103,076)** | **CKD patients**  **(n = 4,654)** | **Non-CKD patients**  **(n = 98,422)** | ***P*-value** |
| --- | --- | --- | --- | --- |
| Age in years, mean (SD, min–max) [years] | 70 (12, 20–105) | 75 (9, 25–98) | 70 (12, 20–105) | <0.001 |
| <65, n (%) | 27,380 (26.6) | 606 (13.0) | 26,774 (27.2) | <0.001 |
| 65–74, n (%) | 36,424 (35.3) | 1,575 (33.8) | 34,849 (35.4) |  |
| ≥75, n (%) | 39,272 (38.1) | 2,473 (53.1) | 36,799 (37.4) |  |
| Sex, n (%) [female] | 45,623 (44.3) | 1,381 (29.7) | 44,242 (45.0) | <0.001 |
| Cancer type, n (%) |  |  |  | <0.001 |
| Colon cancer | 55,025 (53.4) | 2,457 (52.8) | 52,568 (53.4) |  |
| Gastric cancer | 27,206 (26.4) | 1,331 (28.6) | 25,875 (26.3) |  |
| NSCLC | 12,152 (11.8) | 678 (14.6) | 11,474 (11.7) |  |
| Breast cancer | 8,693 (8.4) | 188 (4.0) | 8,505 (8.6) |  |
| Adjuvant chemotherapy, n (%) | 65,428 (63.5) | 1,936 (41.6) | 63,492 (64.5) | <0.001 |
| Postoperative length of stay, median (IQR) [days] | 11 (8–16) | 13 (10–21) | 11 (8–16) | <0.001 |
| Duration of adjuvant chemotherapy, median (IQR) [days] | 154 (98–228) | 147 (66–229) | 154 (99–228) | <0.001 |
| Barthel Index score, n (%) |  |  |  | <0.001 |
| 100 | 91,489 (88.8) | 3,802 (81.7) | 87,687 (89.1) |  |
| 60–95 | 7,606 (7.4) | 555 (11.9) | 7,051 (7.2) |  |
| <60 | 3,981 (3.9) | 297 (6.4) | 3,684 (3.7) |  |
| Comorbidity, n (%) | 32,177 (31.2) | 2,766 (59.4) | 29,411 (29.9) | <0.001 |
| Cardiovascular disease | 14,409 (14.0) | 1,322 (28.4) | 13,087 (13.3) | <0.001 |
| Diabetes mellitus | 19,541 (19.0) | 1,929 (41.4) | 17,612 (17.9) | <0.001 |
| Liver disease | 3,782 (3.7) | 226 (4.9) | 3,556 (3.6) | <0.001 |
| Hospital type, n (%) |  |  |  | <0.001 |
| Non-designated hospital | 21,743 (21.1) | 1,118 (24.0) | 20,625 (21.0) |  |
| Designated hospital | 81,333 (78.9) | 3,536 (76.0) | 77,797 (79.0) |  |
| Year of diagnosis, n (%) |  |  |  | <0.001 |
| 2016 | 22,598 (21.9) | 889 (19.1) | 21,709 (22.1) |  |
| 2017 | 22,311 (21.6) | 967 (20.8) | 21,344 (21.7) |  |
| 2018 | 28,198 (27.4) | 1,345 (28.9) | 26,853 (27.3) |  |
| 2019 | 29,969 (29.1) | 1,453 (31.2) | 28,516 (29.0) |  |

Abbreviations: SD, standard deviation; min, minimum; max, maximum; IQR, interquartile range; NSCLC, non-small-cell lung cancer; CKD, chronic kidney disease.

**Supplementary Table 2**. Factors related to the performance of adjuvant chemotherapy in all patients in hospitals in which hemodialysis could be performed (n = 103,076)

| Characteristics | Unadjusted odds ratio (95% CI) | P-value | Adjusted odds ratio (95% CI)^a^ | P-value |
| --- | --- | --- | --- | --- |
| CKD |  |  |  |  |
| No | Reference |  | Reference |  |
| Yes | 0.39 (0.37–0.42) | <0.001 | 0.51 (0.47–0.54) | <0.001 |
| Age [years] |  |  |  |  |
| <65 | Reference |  | Reference |  |
| 65–74 | 0.56 (0.54–0.58) | <0.001 | 0.62 (0.59–0.65) | <0.001 |
| ≥75 | 0.12 (0.11–0.12) | <0.001 | 0.15 (0.14–0.15) | <0.001 |
| Sex |  |  |  |  |
| Male | Reference |  | Reference |  |
| Female | 1.02 (1.00–1.05) | 0.08 | 1.04 (1.01–1.07) | 0.02 |
| Barthel Index score |  |  |  |  |
| 100 | Reference |  | Reference |  |
| 60–95 | 0.21 (0.20–0.22) | <0.001 | 0.33 (0.31–0.34) | <0.001 |
| <60 | 0.06 (0.06–0.07) | <0.001 | 0.09 (0.08–0.10) | <0.001 |
| Comorbidity |  |  |  |  |
| No | Reference |  | Reference |  |
| Yes | 0.59 (0.57–0.60) | <0.001 | 0.80 (0.78–0.83) | <0.001 |
| Hospital type |  |  |  |  |
| Non-designated | Reference |  | Reference |  |
| Designated | 1.04 (1.00–1.07) | 0.02 | 0.90 (0.87–0.93) | <0.001 |

^a^Adjusted for age at diagnosis, sex, Barthel Index, comorbidities, and hospital type.

Abbreviations: CI, confidence interval; CKD, chronic kidney disease.
